# Correlates of Metabolic Abnormalities in Bipolar I Disorder at Initiation of Acute Phase Treatment

**DOI:** 10.4306/pi.2009.6.2.78

**Published:** 2009-06-30

**Authors:** Byungsu Kim, Sangeok Kim, Roger S. McIntyre, Hui Joon Park, Seong Yoon Kim, Yeon Ho Joo

**Affiliations:** 1 Department of Psychiatry and Health Promotion Center, Asan Medical Center, University of Ulsan College of Medicine, Seoul, Korea.; 2 Department of Psychiatry, Asan Medical Center, University of Ulsan College of Medicine, Seoul, Korea.; 3 Department of Psychiatry, University of Toronto, and the Mood Disorders Psychopharmacology Unit, University Health Network, Toronto, Ontario, Canada.; 4 Department of Psychiatry, Ulsan University Hospital, University of Ulsan College of Medicine, Ulsan, Korea.

**Keywords:** Bipolar disorder, Hypercholesterolemia, Hyperglycemia, Obesity

## Abstract

**Objective:**

Treatment of bipolar patients is often complicated by metabolic abnormalities such as obesity, diabetes, and dyslipidemia. We therefore evaluated the prevalence of these abnormalities and their correlates, in bipolar I patients, at the time of commencement of pharmacological treatment for acute mood episodes.

**Methods:**

The study cohort consisted of 184 bipolar I patients hospitalized for treatment of acute mood episodes. Socio-demographic and clinical variables were noted and metabolic parameters, including body mass index, fasting plasma glucose, fasting total cholesterol, and current treatment(s) for diabetes and/or dyslipidemia were measured before initiating medication(s).

**Results:**

Fifty-six (30.4%) subjects met our criteria for obesity; 80 (43.5%) had hyperglycemia, with 8 (4.3%) receiving anti-diabetic medication; and 38 (20.7%) had hypercholesterolemia, with 2 (1.1%) receiving cholesterol-lowering agents. We found that male sex (χ^2^=5.359, p=0.021), depressed or mixed state versus manic state (χ^2^=4.302, p=0.038), and duration of illness (t=2.756, p=0.006) were significantly associated with obesity. Older age (t=3.668, p<0.001), later age of disease onset (t=2.271, p=0.024), and lower level of educational attainment (β=-0.531, p=0.001) were associated with hyperglycemia.

**Conclusion:**

Our finding that metabolic abnormalities are prevalent when initiating acute pharmacological treatment in bipolar I patients indicates that these factors should be integrated into treatment plans at the onset of disease management.

## Introduction

Treatment of patients with bipolar disorder is often complicated by metabolic abnormalities including obesity, diabetes, and dyslipidemia. The prevalence of obesity in patients with bipolar disorder has been reported to range from 20% to 35%, significantly higher than in the general population.^1^-^5^ In addition, the prevalence of type II diabetes mellitus in patients with bipolar I disorder has been reported to range from 10% to 26%, as much as threefold higher than matched estimates in the general population.^6^-^8^ Moreover, abnormalities in cholesterol and fatty acid metabolism may be associated with bipolar disorders.^9^-^11^ Furthermore, 30% to 49% of bipolar patients have been found to meet the criteria for metabolic syndrome, which is characterized by dysfunctions in glucose and lipid metabolism.^12^,^13^

The burden of metabolic syndrome is not limited to medical problems, such as type 2 diabetes mellitus, cardiovascular disease, and stroke,^14^-^16^ but can also affect mental illness presentation, course, and response to treatment. For example, obesity in bipolar patients has been correlated with a greater number of lifetime depressive and manic episodes and a greater risk of developing an affective recurrence.^4^ Recent work has shown that bipolar patients with diabetes mellitus had a greater number of lifetime psychiatric hospitalizations than did non-diabetic bipolar patients.^17^ Bipolar patients who met the criteria for metabolic syndrome were more likely to report a lifetime history of suicide attempts than those without the metabolic syndrome.^12^ Thus, preventing metabolic dysregulation might not only decrease medical illness and mortality in bipolar patients, but may also improve course of illness in bipolar disorder.

Decisions regarding pharmacological treatment are increasingly influenced by concerns regarding metabolic risks associated with individual medications. For example, treatment with atypical antipsychotics has been associated with clinically significant weight gain and obesity, as well as with hyperglycemia, exacerbation of pre-existing diabetes or development of type 2 diabetes.^18^ Polypharmacy is a common practice in bipolar disorder. Furthermore, combinations of atypical antipsychotics and mood stabilizers may result in additional risk for obesity and associated metabolic abnormalities in bipolar patients.^19^ The hazards posed by metabolic abnormalities as well as the risk for further disruption of metabolic parameters with psychiatric medication invites the need for a priori consideration of metabolic issues in all bipolar patients.

The baseline pretreatment prevalence of metabolic abnormalities holds promise to improve patient outcome visà-vis risk stratification and appropriate medication assignment. Hitherto, most studies documenting metabolic profiles in bipolar patients have most often included individuals who have received complex pharmacological regimens prior to evaluation. In this study we extend knowledge regarding metabolic abnormalities in bipolar individuals by reporting on the prevalence of obesity, hyperglycemia, and hypercholesterolemia, and their clinical correlates, in bipolar I patients prior to the initiation of pharmacological treatment for an acute mood episode.

## Methods

The study cohort consisted of 184 bipolar I patients hospitalized at a university hospital in Seoul, Korea, for treatment of acute mood episodes between January 2005 and December 2006. Subjects were between 19 and 64 years of age. All patients were diagnosed according to the criteria of the Diagnostic and Statistical Manual of Mental Disorders, fourth edition (DSM-IV) by two or more experienced psychiatrists. For patients admitted more than once during the study period, data from the index admission were used. The institutional review board of the Asan Medical Center, Ethics Committee, approved this study, and waived the requirement for informed consent because measurements of weight and height and assessments adapted in this study were part of routine clinical practice.

Socio-demographic and clinical variables were collected, and metabolic parameters, including body mass index (BMI), fasting plasma glucose (FPG), and fasting total cholesterol, were measured before initiating medication (s). Current treatment(s) for diabetes and/or dyslipidemia was confirmed by review of each patient's medical records. Physical data included body weight and height, with BMI calculated as weight in kilograms divided by height in meters squared. Obesity was defined according to the criterion and classification in Japan and Asia-Oceania,^20^ as BMI over 25 kg/m^2^. This criterion is considered appropriate for Asians whose main energy intake comes from carbohydrates, and has been adapted for Koreans. Biochemical parameters were checked on the morning of the day following admission, with the patient in the fasting state. Hyperglycemia was defined as FPG ≥110 mg/dL and hypercholesterolemia as ≥200 mg/dL.

To assess the correlation of obesity, hyperglycemia, and hypercholesterolemia with socio-demographic and clinical factors, we recorded patient age, sex, marital status, years of education, occupation, socioeconomic status, age at onset of illness, duration of illness, number of previous hospitalizations, and history of previous psychiatric medications. Socio-demographic and clinical variables are reported as means and standard deviations for continuous variables and frequency/percentage for non-continuous variables. Chi-square or Fisher's exact tests for sex, mood state, history of psychotropic medications, occupation, socio-economic status and marital status were used to assess differences between two groups (obese vs. non-obese; hyperglycemic vs. normal FPG; hypercholesterolemic vs. normal cholesterol). Continuous variables, including age, age of disease onset, duration of illness, and number of previous hospitalizations, were compared using Student's t-test. Logistic regression analysis, using the two potential correlates as independent variables, was used to determine if there were significant correlations between educational levels and the presence or absence of metabolic abnormalities. All statistical analyses were performed with Statistical Package for Social Science (SPSS) version 12.0 for Windows, with statistical significance defined as p<0.05.

## Results

The socio-demographic and clinical characteristics of study subjects are presented in Table 1. The mean patient age was 38.0±13.2 years, and 96 (52.2%) were female. The mean age of disease onset was 30.8±11.7 years and the mean duration of illness was 8.2±8.4 years. Of the 184 subjects, 128 (69.6%) were manic, 48 (26.1%) were depressed, and 8 (4.3%) were mixed at the time of hospitalization. Prior medications used before the index hospitalizations are listed in Table 2. Although most patients were taking more than one medication, 70 (38.0%) had never been prescribed psychotropic medications. The most frequently prescribed antipsychotics were olanzapine (26.1%) and risperidone (25.5%), whereas the most frequently prescribed mood stabilizers were lithium (31.0%) and divalproex (29.9%).

We found that 56 (30.4%) bipolar patients met the Asian criterion for obesity (BMI ≥25 kg/m^2^)(Table 3); 80 (43.5%) had hyperglycemia (FPG ≥110 mg/dL), with 8 (4.3%) taking anti-diabetic agents; and 38 (20.7%) had hypercholesterolemia (≥200 mg/dL), with 2 (1.1%) on cholesterol-lowering agents (Table 2). There was a significant difference in the prevalence of obesity between male and female subjects (χ^2^=5.359, p=0.021), but no other significant differences related to sex (Table 3).

Table 4 shows comparisons of socio-demographic and clinical characteristics between groups sorted according to the presence of each metabolic variable. When we compared obese and non-obese subjects, we found that male sex (χ^2^=5.359, p=0.021), episode polarity, i.e. depressed or mixed rather than manic state (χ^2^=4.302, p=0.038), and duration of illness (t=2.756, p=0.006) were significantly associated with obesity. Although the level of education did not differ significantly in obese and non-obese subjects, less-educated subjects tended to have a higher prevalence of obesity (p=0.054). Older age (t=3.668, p<0.001), later age of disease onset (t=2.271, p=0.024), and lower level of education (β=-0.531, p=0.001) were significantly associated with hyperglycemia. We found no statistically significant variable associated with hypercholesterolemia (data not shown). When we sorted groups according to history of usage of psychotropic medications (i.e. antipsychotics, mood stabilizers, and antidepressants), we found no significant differences in the frequencies of abnormalities in metabolic parameters (Table 5).

Of the variables investigated, multiple logistic regression analysis showed that sex (p=0.007) and episode polarity (p=0.011) were independently associated with obesity, and that age (p=0.005) and educational level (p=0.012) were independently associated with the presence of hyperglycemia or anti-diabetic medication usage.

## Discussion

Our analysis indicates that disparate metabolic variables e.g. obesity, hyperglycemia, and hypercholesterolemia, were highly prevalent in a large sample of patients (n=184) with bipolar I disorder at the time of hospitalization. As the prevalence of metabolic problems is significantly higher in bipolar patients than in the general population, pharmacologic treatment should be implemented only after consideration of metabolic risk factors. In particular, bipolar patients beginning pharmacological treatment for acute mood episodes may be more vulnerable to metabolic derangements, because significant weight gain has been shown to occur during acute treatment with atypical antipsychotics in combination with mood stabilizers.^19^ Therefore, it is clinically important to determine whether metabolic risks are present at the initiation of acute treatment, as well as to measure the clinical and socio-demographic variables associated with metabolic risks in bipolar patients.

Our findings need to be considered in the context of extant data. For example, epidemiological and clinical studies indicate that the prevalence of obesity in bipolar individuals is approximately 20% to 35%.^1^-^5^ Our observation that 30.4% of patients in this cohort were obese at the start of treatment is in line with previous reports. We observed significant associations between obesity and male sex, episode polarity i.e. depressed or mixed, and longer duration of illness. Multiple logistic regression analysis showed that male sex and depressive or mixed state were significant independent factors associated with obesity. These correlations are consistent with results reporting that male sex^21^ and depressive symptoms^3^,^4^ were related to obesity in bipolar patients. In addition, years of education, economic status, and ethnicity have been found to be associated with obesity.^4^,^21^ Although we found that the prevalence of obesity tended to be higher in subjects with a lower level of education, this association was not statistically significant.

The overall prevalence of hyperglycemia in our study cohort was significantly elevated at 43.5%. Data from studies evaluating metabolic syndrome in Caucasian patients with bipolar disorder reported a prevalence of hyperglycemia (i.e. FPG ≥110 mg/dL), ranging from 8.0% to 16.9%.^12^,^22^-^25^ As FPG >110 mg/dL has been reported to be the most specific factor (95.2%) in correctly identifying the presence of metabolic syndrome,^26^ the high proportion of our patients with elevated FPG is note worthy. Heterogeneity in study outcomes regarding hyperglycemia may be due to differences between studies in sample composition, definitions of hyperglycemia, and sampling procedures. For example, some Asian populations are differentially affected by insulin resistance and diabetes mellitus.^27^,^28^ Although we did not measure cortisol concentrations, hyper-cortisolemia may be associated with hyperglycemia, as acute mood symptoms may be preceded and accompanied by increases in adrenocorticotropic hormone levels. The disturbances in cortisol regulation seen in bipolar disorder play a role in the development of core manifestations of metabolic syndrome, such as insulin resistance, hyperglycemia, obesity, and dyslipidemia.^29^

Subjects presenting with hyperglycemia or taking anti-diabetic medications were significantly older (42.0±12.8 years) than those without hyperglycemia (35.0±12.8 years). Aging is accompanied by a loss of muscle mass and by an increase in body fat, which may lead to increased insulin resistance and subsequent metabolic syndrome.^30^ Moreover, diabetic bipolar patients have been reported to be significantly older than non-diabetic bipolar patients.^13^,^17^,^31^ The prevalence of hyperglycemia was significantly associated with a lower level of education in bipolar patients. Although we could not determine a causal relationship between education and hyperglycemia, an unhealthy life style, including increased intake of carbohydrates and sweets, may be contributory.

We also found that 20.7% of our subjects had increased total cholesterol levels (≥200 mg/dL). To our knowledge, there have been no studies specifically addressing the frequency of abnormal cholesterol levels in bipolar patients at the time of acute mood episodes. Combined with the high frequency of obesity and hyperglycemia, our finding that 20% of patients had hypercholesterolemia warrants the need for attention when treating bipolar patients with medications that can disrupt metabolic parameters.

As atypical antipsychotics have been reported to be associated with a high prevalence of metabolic abnormalities,^32^,^33^ bipolar patients prescribed these medications are more likely to exhibit a greater frequency of metabolic disturbances, including obesity, hyperglycemia, and hypercholesterolemia. However, we did not observe a significant association between the uses of psychotropic medications before entering acute phase treatment with interferences in these parameters. Additionally, subgroup analyses of patients treated with some atypical antipsychotics, i.e. olanzapine, risperidone, and quetiapine, did not reveal an independent association between use of these medications and obesity, hyperglycemia, or hypercholesterolemia (data not shown). These unexpected results may in part be due to limitations in adherence to medication. In particular, bipolar patients exhibit high rates of nonadherence with prescribed treatments both pharmacological and psychosocial. In addition, because the effects of medication may compound inherent risk factors for metabolic syndrome, the effects of medication cannot fully account for the myriad metabolic abnormalities observed in our patients.

Our study had several methodological limitations. First, we did not collect detailed information on health habits, such as smoking, use of drugs and alcohol, excessive caloric and cholesterol intake, and physical inactivity, all of which may contribute to the metabolic abnormalities observed. A second limitation was the cross-sectional design of our study which precludes us from establishing causal relationships among variables. Third, our sample was relatively small (n=184) and derived from a single university hospital which may not be representative of outpatients with bipolar disorder and/or other individuals with less complex illness presentations. A fourth limitation is that our study did not include other metabolic parameters, such as levels of triglyceride and low-and high-density lipoprotein cholesterol, which are important constituents of metabolic syndrome. Lastly, we did not control for baseline indices of illness severity or length of psychotropic drug treatment. In addition, our subjects consisted of drug-naïve patients and those who have taken psychotropic medications before enrolling this study, which could affect the results of our study. The finding that there was no significant association between the uses of psychotropic medications before entering acute phase treatment and the interferences of metabolic parameters should be interpreted by considering this particular methodological limitation.

In summary, metabolic abnormalities are prevalent in bipolar individuals at the time of initiating pharmacological treatment for an acute episode requiring hospitalization. The high prevalence of metabolic abnormalities as well as the hazards posed by their occurrence invites the need for practitioners to carefully screen all bipolar patients for risk factors related to metabolic abnormalities as well as careful clinical surveillance for incident disturbances in any metabolic parameter. The selection and sequencing of psychotropic agents in managing bipolar patients needs to be informed by possible metabolic disruption. The incorporation of psychosocial treatment strategies that emphasize educational aspects related to metabolic risk and medical comorbidity holds promise to reduce the burden of illness and improve outcomes in bipolar disorder.

## Figures and Tables

**TABLE 1:**
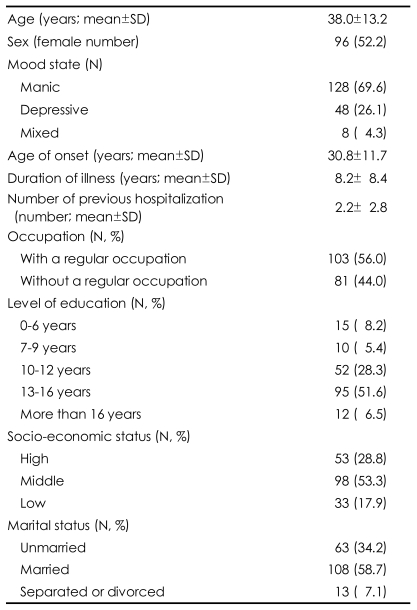
Socio-demographic and clinical characteristics of bipolar I patients (N=184) at initiating acute phase treatment (%)

**TABLE 2:**
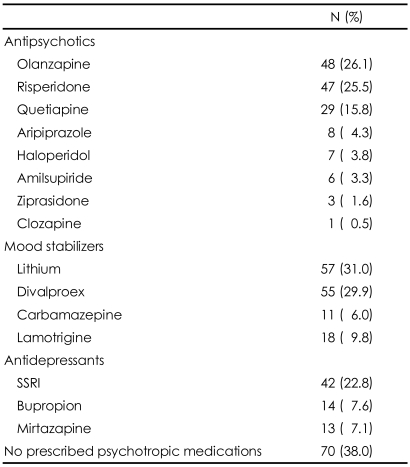
Psychotropic medications used before entering acute phase treatment

If more than 2 medications had been used in a patient, each medication was counted separately. SSRI: selective serotonin reuptake inhibitor

**TABLE 3:**
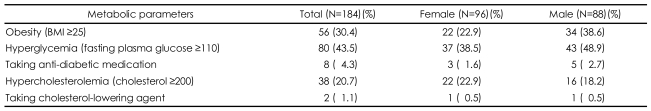
Prevalence of metabolic abnormalities in bipolar I patients

BMI: body mass index

**TABLE 4:**
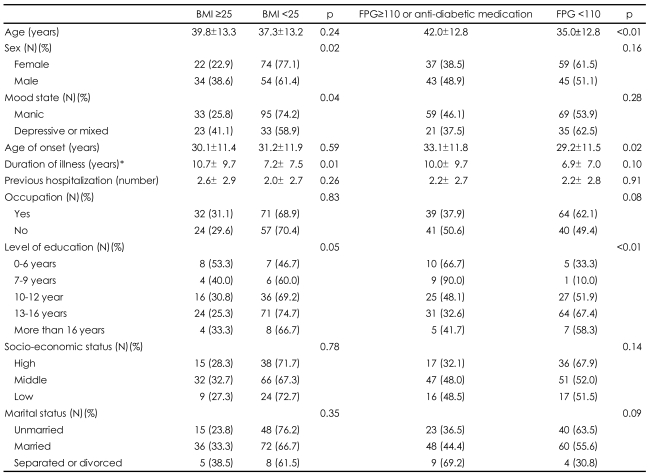
Correlates of metabolic abnormalities in bipolar I patients

^*^t-test after log transformation. BMI: body mass index, FPG: fasting plasma glucose

**TABLE 5:**
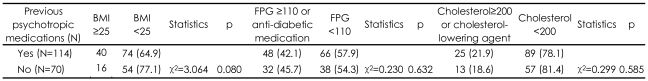
Comparison of patients who have taken psychotropic medication(s) before entering acute treatment and those who have not

BMI: body mass index, FPG: fasting plasma glucose
